# Urinary Extracellular Vesicle Signatures as Biomarkers in Prostate Cancer Patients

**DOI:** 10.3390/ijms26146895

**Published:** 2025-07-18

**Authors:** Sigrun Lange, Darryl Ethan Bernstein, Nikolay Dimov, Srinivasu Puttaswamy, Ian Johnston, Igor Kraev, Sarah R. Needham, Nikhil Vasdev, Jameel M. Inal

**Affiliations:** 1 Pathobiology and Extracellular Vesicles Research Group, School of Life Sciences, University of Westminster, London W1W 6UW, UK; s.lange@westminster.ac.uk; 2 Hertfordshire and Bedfordshire Urological Cancer Centre, Department of Urology, Lister Hospital, East and North Hertfordshire Teaching NHS Trust, Stevenage, Hertfordshire SG1 4AB, UK; darryl.bernstein@nhs.net (D.E.B.); nikhil.vasdev@nhs.net (N.V.); 3 Department of Engineering and Technology, School of Physics, Engineering & Computer Science, University of Hertfordshire, Hatfield AL10 9EU, UK; n.dimov@herts.ac.uk (N.D.); s.puttaswamy@herts.ac.uk (S.P.); i.d.johnston@herts.ac.uk (I.J.); 4 Electron Microscopy Suite, Faculty of Science, Technology, Engineering and Mathematics, Open University, Milton Keynes MK7 6AA, UK; igor.kraev@open.ac.uk; 5 UKRI: Science & Technology Facilities Council, Central Laser Facility, Rutherford Appleton Laboratory, Oxfordshire OX11 0QX, UK; sarah.needham@stfc.ac.uk; 6 School of Life and Medical Sciences, University of Hertfordshire, Hatfield AL10 9EU, UK; 7 Cell Communication in Disease Pathology, School of Human Sciences, London Metropolitan University, London N7 8DB, UK; 8 Biosciences Research Group, School of Life and Medical Sciences, University of Hertfordshire, Hatfield AL10 9EU, UK

**Keywords:** prostate cancer, urinary biomarkers, extracellular vesicles, liquid biopsy, proteome, gene ontology, KEGG, apoptosis, immunity, stress response

## Abstract

Urinary extracellular vesicles (U-EVs) are gaining increasing interest as non-invasive liquid biopsy tools for clinical use. Prostate cancer (PCa) is amongst the highest cancer-related cause of death in men, and therefore, the identification of non-invasive robust biomarkers is of high importance. This study assessed U-EV profiles from individuals affected by PCa at Gleason scores 6–9, compared with healthy controls. U-EVs were characterised and assessed for proteomic cargo content by LC-MS/MS analysis. The U-EV proteomes were compared for enrichment of gene ontology (GO), KEGG, and Reactome pathways, as well as disease–gene associations. U-EVs ranged in size from 50 to 350 nm, with the majority falling within the 100–200 nm size range for all groups. U-EV protein cargoes from the PCa groups differed significantly from healthy controls, with 16 protein hits unique to the GS 6–7 and 88 hits to the GS 8–9 U-EVs. Pathway analysis showed increased enrichment in the PCa U-EVs of biological process GO (5 and 37 unique to GS 6–7 and GS 8–9, respectively), molecular function GO (3 and 6 unique to GS 6–7 and GS 8–9, respectively), and cellular component GO (10 and 22 unique to GS 6–7 and GS 8–9, respectively) pathways. A similar increase was seen for KEGG pathways (11 unique to GS 8–9) and Reactome pathways (102 unique to GS 8–9). Enrichment of disease–gene associations was also increased in the PCa U-EVs, with highest differences for the GS 8–9 U-EVs (26 unique terms). The pathway enrichment in the PCa U-EVs was related to several key inflammatory, cell differentiation, cell adhesion, oestrogen signalling, and infection pathways. Unique GO and KEGG pathways enriched for the GS 8–9 U-EVs were associated with cell–cell communication, immune and stress responses, apoptosis, peptidase activity, antioxidant activity, platelet aggregation, mitosis, proteasome, mRNA stability oxytocin signalling, cardiomyopathy, and several neurodegenerative diseases. Our findings highlight U-EVs as biomarkers to inform disease pathways in prostate cancer patients and offer a non-invasive biomarker tool for clinical use.

## 1. Introduction

Prostate cancer (PCa) is the second leading cancer-related death in men globally, with approximately 1.4 million new cases and 400,000 deaths annually [1]. As the current means of PCa diagnosis is by invasive transrectal ultrasound-guided biopsy [2], there is an important need to identify non-invasive and usable biomarkers.

Extracellular vesicles (EVs) play key roles in cellular communication via transfer of their cargo proteins and other cargo, including non-coding RNAs and genetic cargoes, between cells and can be isolated from most body fluids, including urine [3]. This places EVs as ideal non-invasive biomarkers. Urinary extracellular vesicles (U-EVs) are gaining increasing interest as disease biomarkers and as non-invasive liquid biopsy tools for clinical use [4,5,6,7]. U-EVs may mirror physiological and pathological conditions in urothelial and prostate tissue and indicate molecular processes [3]. Studies have indicated that U-EVs may be indicative for risk evaluation in PCa, beyond the use of prostate-specific antigen [8]. Recent studies have, for example, evaluated various micro-RNA cargoes [9] and explored various U-EV protein cargoes [10,11,12]. However, results from diverse studies have also highlighted the need for further investigations to fully understand the role of U-EVs and their associated markers for clinical diagnostics. It is therefore of great interest for clinical applications to further our current understanding of how U-EVs can be used as biomarkers, and how they can provide accurate information on cancer-related processes.

This study aimed at screening U-EVs from PCa patients at Gleason scores (GSs) 6 to 9, compared to healthy controls, for identification of changes in proteomic signatures that may be indicative of PCa grading. The Gleason grading system is a histologic evaluation of tumour architecture and ranges from 2 to 10, with 6 being the lowest score currently assigned and indicative of intermediate prognosis [13,14]. Our aim was to assess putative U-EV-associated biomarkers associated with PCa and furthermore to distinguish U-EV signatures between lower and higher GS, compared with healthy controls. We hypothesise that U-EVs have increased proteomic content with higher GS and are associated with cancer-related pathways.

## 2. Results

### 2.1. Characterisation, Quantification, and Size Profiling of Urinary EVs from Healthy Controls and Prostate Cancer Patients

**Figure 1:**
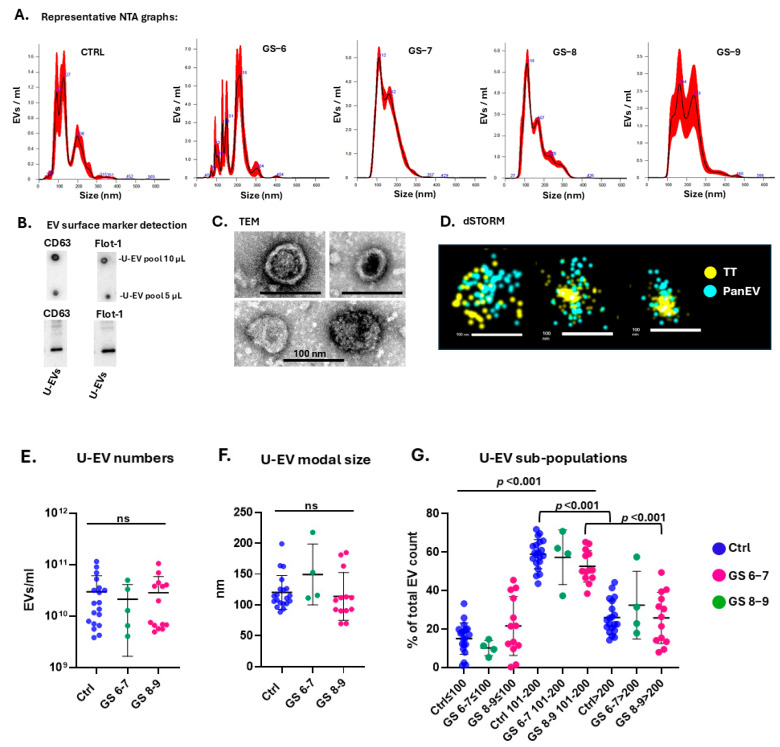
Urinary EV characterisation and quantification. (**A**) NTA—showing representative size distribution graph for U-EVs from control, GS−6, GS−7, GS−8, and GS−9 groups, respectively (the black line represents the mean, and the red line represents the standard error for the mean, SEM). (**B**) Surface marker detection for CD63 and flotillin-1 of U-EVs by dot blot and Western blotting. (**C**) TEM images of U-EVs; scale bar = 100 nm for all. (**D**) dSTORM imaging of U-EVs (yellow = tetraspanin trio marker CD9+, CD63+ and CD81+; blue = pan-EV marker); scale bar = 100 nm for all. (**E**) U-EV quantification per sample group, showing controls (Ctrl) and Gleason scores 6−7 and 8−9, respectively. (**F**) U-EV modal size for the three sample groups. (**G**) U-EV subpopulations for the three sample groups; n = 20 for controls; n = 6 for GS 6−7, and n = 13 for GS 8−9. The error bars represent mean and standard deviation (SD); ns means not significant; *p*-values are indicated.

Characterisation of U-EVs was carried out by NTA (with representative NTA graphs per sample group shown in Figure 1A), for EV surface marker detection of CD63 and flotillin-1 by dot blot and Western blotting (Figure 1B), and for morphology by transmission electron microscopy (TEM; Figure 1C). U-EVs were also assessed by direct stochastic optical reconstruction microscopy (dSTORM) (Figure 1D), confirming positive labelling with tetraspanin trio (CD9, CD63, CD81) and pan-EV stain (Figure 1D). The concentration of EVs per urine sample showed considerable individual variation (n = 39 across all three sample groups), with an average concentration of 2.5 × 10^10^/mL, ranging from 3.82 × 10^9^/mL to 1.15 × 10^11^/mL. No significant differences were observed in EV numbers per ml of concentrated urine sample between the three groups (Figure 1E). EV modal size ranged from 70 to 217 nm, with no significant differences observed between the three groups (Figure 1F). When analysing EV subpopulations according to size distribution, the highest proportion of EVs fell within the 101–200 nm size range (medium-EVs) for all three sample groups (Figure 1G).

### 2.2. Protein–Protein Interaction (PPI) Network and Pathway Enrichment Analysis for Urinary EV Proteome Cargoes

**Figure 2:**
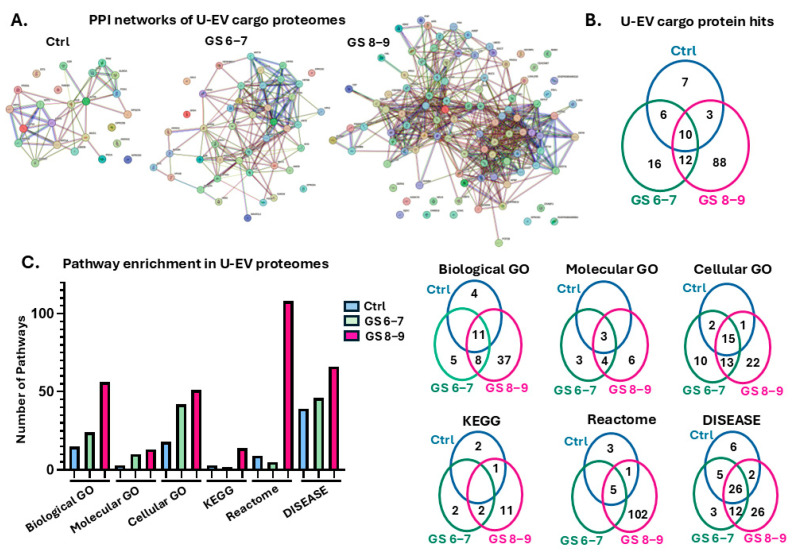
Protein–protein interaction (PPI) networks and pathway enrichment analysis for the U-EV proteomes. (**A**) PPI networks for control (Ctrl; PPI enrichment *p*-value: 3.93 × 10^−12^), Gleason score 6–7 (GS 6−7; PPI enrichment *p*-value: <1.0 × 10^−16^), and Gleason score 8−9 (GS 8−9; PPI enrichment *p*-value: <1.0 × 10^−16^), with individual protein hits represented by the coloured nodes. (**B**) A Venn diagram summarising the number of shared and unique protein hits in the U-EVs of each group. (**C**) Pathway enrichment analysis of the U-EV proteomes for gene ontology (GO) biological process, molecular function, and cellular component pathways, KEGG pathways, Reactome pathways, and disease–gene associations (DISEASE), summarising the number of shared and unique pathways for each analysis in the Venn diagrams. For a full list of protein hits in all three groups, see Supplementary Table S1; for full lists of enriched pathways in all three groups, see Supplementary Tables S2–S7.

The U-EV cargoes were assessed for proteomic content by LC-MS/MS for the three groups (Ctrl n = 20; GS 6–7 n = 6; GS 8–9 n = 13) with proteomic hits identified per group, as listed in Supplementary Table S1. The proteomic U-EV cargoes as identified by LC MS/MS were analysed in STRING for protein–protein interactions (PPIs), and networks were generated, showing more significant *p*-values for the PPI enrichment of the PCa U-EVs, alongside considerably more protein targets in the GS 8–9 U-EVs, compared with the GS 6–7 U-EVs (Figure 2A). A summary of unique and shared protein hits between the groups is provided in the Venn diagram in Figure 2B. Gene ontology (GO) pathway analysis showed considerable differences between the healthy control and PCa U-EV proteomes, with 15, 24, and 56 biological process GO; with 3, 10 and 13 molecular function GO; and with 18, 40 and 51 cellular component GO pathways enriched in the control, GS 6–7, and GS 8–9 U-EV proteomes, respectively (Figure 2C). In the U-EVs, 3, 2, and 14 KEGG pathways were enriched in the control, GS 6–7, and GS 8–9 proteomes, respectively (Figure 2C). When assessing Reactome pathways, 9, 5, and 108 pathways were enriched in the control, GS 6–7, and GS 8–9 U-EV proteomes, respectively (Figure 2C). For disease–gene associations (DISEASES), 39, 46, and 66 terms were enriched in the control, GS 6–7, and GS 8–9 U-EV proteomes, respectively (Figure 2C). The numbers of shared and unique enriched pathways and terms are summarised in the Venn diagrams in Figure 2C.

**Figure 3:**
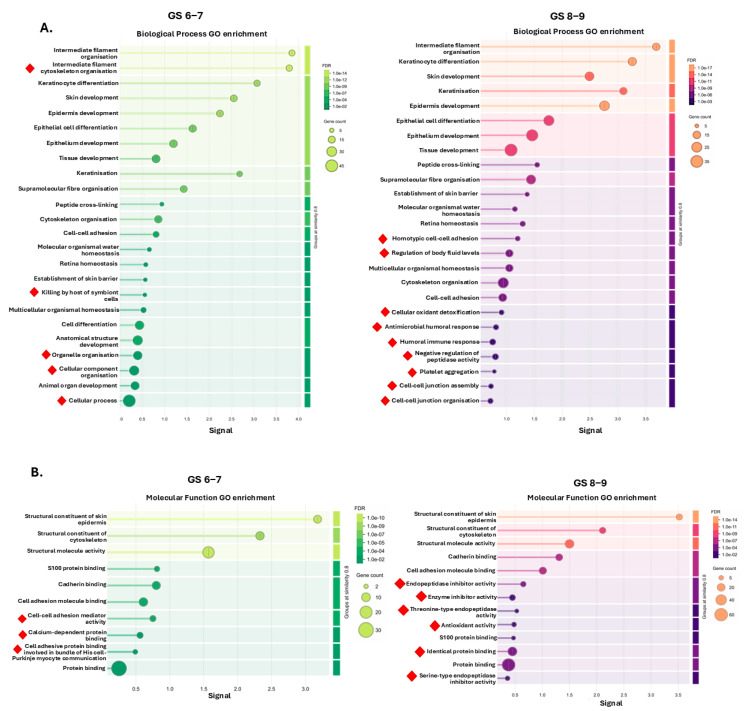

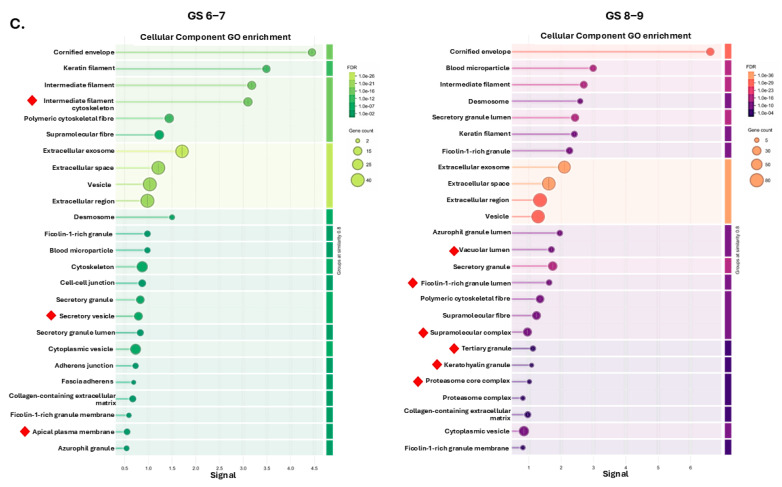
Gene ontology (GO) analysis of the U-EV proteomes. The top 25 enriched pathways identified for the U-EV proteomes of the Gleason score 6−7 and Gleason score 8−9 groups are shown, respectively (or fewer, if fewer than 25 pathways were identified for the group). (**A**) Biological process GO; (**B**) molecular function GO; (**C**) cellular component GO pathways. The graphs show Signal on the *x*-axis and false discovery rate (FDR) on the *y*-axis. Gene count indicates the number of proteins associated with the term. The red diamonds indicate terms which are unique to the respective GS group only, excluding shared terms.

For all pathway enrichment analyses, the top 25 pathways per GS group (or fewer, if fewer than 25 were identified) are presented in Figure 3 for biological process GO (Figure 3A), molecular function GO (Figure 3B), and cellular component GO pathways (Figure 3C). Within the top 25 enriched biological GO pathways, the unique ones for the GS 6–7 U-EV proteome were the following: intermediate filament cytoskeletal organisation; killing by host of symbiont cells; organelle organisation; cellular component organisation; and cellular process. For the GS 8–9 group, the unique ones within the top 25 enriched biological GO pathways included the following: homotypic cell–cell adhesion; regulation of body fluid levels; cellular oxidant detoxification; antimicrobial humoral response; humoral immune response; negative regulation of peptidase activity; platelet aggregation; and cell–cell junction assembly and organisation (Figure 3A). Molecular function GO pathways unique to the GS 6–7 U-EV proteome were as follows: cell–cell adhesion mediator activity; calcium-dependent protein binding; and cell-adhesive protein binding involved in bundle of His cell–Purkinje myocyte communication. For the GS 8–9 group, unique molecular function GO enrichment was related to endopeptidase inhibitor activity; enzyme inhibitor activity; threonine-type endopeptidase activity; antioxidant activity; identical protein binding; and serine-type endopeptidase inhibitor activity (Figure 3B). For cellular component GO pathway enrichment, unique pathways out of the top 25 enriched for the GS 6–7 U-EV proteome were as follows: intermediate filament cytoskeleton; secretory vesicle; and apical plasma membrane. For the GS 8–9 group, the unique ones within the top 25 pathways were as follows: vascular lumen; ficolin-1-rich granule lumen; supramolecular complex; tertiary granule; keratohyalin granule; and proteasome core complex. The full lists for all GO pathways identified in the three groups are provided in Supplementary Tables S2–S4.

**Figure 4:**
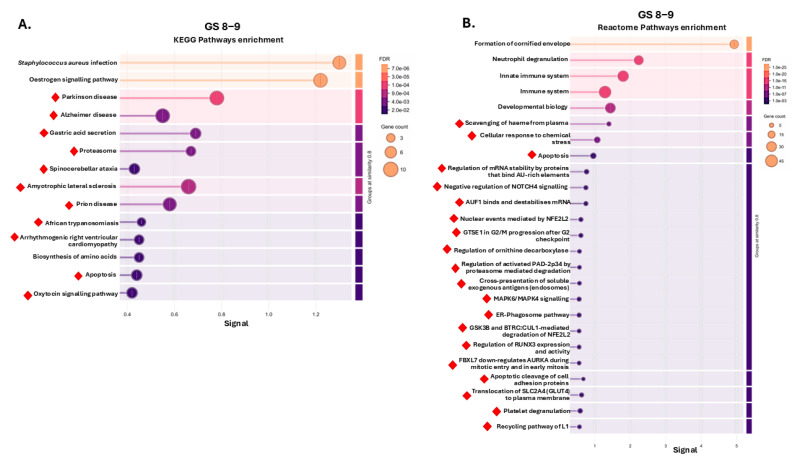
KEGG and Reactome pathway enrichment analysis for the U-EV proteome at GS 8–9. (**A**) All KEGG pathways; (**B**) top 25 Reactome pathways. The graphs show Signal on the *x*-axis and false discovery rate (FDR) on the *y*-axis. Gene count indicates the number of proteins associated with the term. The red diamonds indicate terms which are unique to the GS 8–9 cancer group only and not shared with the other groups; no unique terms were associated with the control or GS 6–7 groups.

Similarly, all the KEGG pathways identified for the GS 8–9 U-EV proteome are shown in Figure 4A, with unique pathways associated with gastric acid secretion; proteasome; apoptosis; oxytocin signalling; cardiomyopathy; and several neurological diseases. While no unique pathways were identified for the GS 6–7 group, the shared pathways with the GS 8–9 group were oestrogen signalling and *S. aureus* infection. The U-EV proteome of the control group had two unique enriched KEGG pathways (glycolysis/gluconeogenesis and carbon metabolism) and one shared KEGG pathway with the GL 8–9 group (biosynthesis of amino acids). A full list of KEGG pathways for all three groups is provided in Supplementary Table S5. Figure 4B shows the top 25 Reactome pathways identified for the U-EV proteome for the GS 8–9 group, with unique pathways relating to the following: scavenging of haeme from plasma; cellular response to chemical stress; apoptosis; regulation of mRNA stability; regulation of NOTCH4 signalling; AUF1 binding; nuclear events mediated by NFE2L2; G2/M progression; regulation of ornithine decarboxylase; proteasome-mediated degradation; exogenous antigen presentation; MAPK signalling; ER-Phagosome; RUNX3 regulation; mitotic and mitosis regulation; and platelet degranulation. While no unique Reactome pathways were associated with the control or GS 6–7 group, the following were shared with both the control and GS 8–9 U-EV proteomes: formation of the cornified envelope; innate immune system; neutrophil degranulation; developmental biology; and cell junction organisation. A full list of Reactome pathways for all three groups is provided in Supplementary Table S6.

For disease–gene association pathways (DISEASES), terms unique to the U-EV proteome of the GS 6–7 group were linked to stomach cancer, duodenal ulcer, and atrophic gastritis. Shared terms for both GS groups included gastrointestinal system cancer, organ system cancer, disease of cellular proliferation, and stomach carcinoma. In addition, there were several DISEASE associations linked to liver, respiratory, genetic, allergic, and autoimmune disease associated with the U-EV proteome of the GS 8–9 group. A full list of all DISEASE terms associated with all three groups is provided in Supplementary Table S7.

## 3. Discussion

This study assessed the profiles and proteomes of U-EVs, comparing healthy control donors to PCa patients with Gleason scores GS 6–7 and GS 8–9. It has been suggested that the characterisation of molecular composition of PCa and its changes with GS may aid faster diagnosis [14]. How such changes may be reflected in U-EV cargoes is therefore of considerable interest. Previous studies have indicated that a higher fraction of PCa-derived EVs can be isolated from urine compared with plasma [4], and proteomic profiling of U-EVs has also shown promise for the identification of breast cancer [15]. Hence, the use of U-EVs in the current study and their proteomic profiling may be of considerable value for PCa disease associations and biomarker discovery. The proteomic analysis of U-EVs here revealed significantly higher amounts of cargo proteins in the PCa U-EVs, also with an increased number of protein hits identified at the higher-score GS group (GS 8–9), compared with the lower-GS-score group (GS 6–7). Specific protein hits which were identified only in the U-EVs of the GS groups may be indicative biomarkers of disease status and are briefly discussed below for both GS groups assessed.

Proteins unique to the U-EVs of the GS 6–7 group included several keratins, corneodesmosin, the cell–cell adhesion protein desmoplakin [16], and uroplakin-2, which is a marker for urothelial and prostate carcinoma [17,18]. Immune- and stress-related proteins included Protein S100-A8, a metastasis-associated protein [19]; T cell receptor alpha joining 56; Mal T-cell differentiation protein 2, which is associated with cancer aggressiveness [20]; programmed cell death protein 6, which has been reported as a prognostic marker for gastric cancers [21]; and peroxiredoxin-1, which is linked to the control of PCa growth [22]. Elongation factor 1-alpha has roles in translation and possible associations with PCa progression [23], while vesicle-fusing ATPase is important for vesicle-mediated transport, with associations in oesophageal squamous cell carcinoma [24], and phosphopyruvate hydratase (enolase) is a key glycolytic enzyme and metalloenzyme with roles in cancer development [25]. In addition, the GS 6–7 U-EV proteomes contained dopamine receptor-interacting protein 4 and brain-specific angiogenesis inhibitor 1-associated protein 2-like protein 2, which is involved in actin filament organisation and has been associated with chemotherapy resistance in advanced gastric cancer [26]. Retinoic acid-induced protein 3 was furthermore unique to the GS 6–7 U-EV proteome and can affect EGFR signalling and has also been linked to tumour suppression [27].

Proteins unique to the U-EVs of the GS 8–9 group included cytoskeleton-related proteins such as actin cytoplasmic 2, F-actin-capping protein subunit beta, tubulin alpha, and AHNAK (desmoyokin), which plays roles in tumour suppression, immune regulation, and calcium homeostasis, as well as in EV release from carcinoma cells [28,29]. Proteins related to the immune response included complement C3, serotransferrin, immunoglobulin gamma-1, and Galectin-7, which is associated with PCa cells [30] and defensin, which is a predictive biomarker of docetaxel response in castration-resistant PCa [31]. Other protein hits included Calmodulin-like protein 3, which has been identified as a target in gastric cancer [32], Serpin A12, Caspase recruitment domain-containing protein 18, Alpha-1-antitrypsin, Fibrinogen alpha, Isoform 2 of 14-3-3 protein sigma, and Cathepsin D, which has been highlighted as a GS-associated biomarker in PCa [33]. Vitamin D-binding protein, also part of the GS 8–9 U-EV proteome, can be an indicative marker of cancer survival [34]. Additional hits were Lysozyme C, Bleomycin hydrolase, Interleukin-36 gamma, AMBP (alpha-1-microglobulin/bikunin precursor), Ganglioside GM2 activator, and Beta globin—which are indicative of cancer cell survival [35]. In addition, proteins related to stress responses and cellular signalling included heat shock cognate 71 kDa protein and heat shock protein beta-1, which are potential therapeutic targets in PCa [36,37], catalase, voltage-dependent anion-selective channel protein1, and Plakophilin 1 and 3—implicated in PCa progression [38,39]. Other targets included Filaggrin-2, peroxiredoxin 6, Calmodulin-like protein 5, TBCEL-TECTA readthrough protein, Desmocollin 1 and 3, and Desmoplakin variant protein—with implications in diagnosis and tumour progression [40]. Metabolic proteins identified as unique to the GS 8–9 UV proteomes included fatty acid-binding protein 5, peroxiredoxin-1, pyruvate kinase, histidine ammonia-lyase, Arginase-1, catalase, phosphoglycerate kinase, and Sialidase-2. In addition, proteins involved in gene transcription, mRNA splicing, and DNA damage repair included [Histone H3]-lysine (36) N-trimethyltransferase, which is associated with epithelial–mesenchymal transition and the invasive properties of PCa [41]. The GS 8–9 UV proteomes furthermore contained epididymis secretory sperm binding protein, the tumour-associated squamous cell carcinoma antigen (SCCA1/SCCA2 fusion protein), CEACAM7—a member of the carcinoembryonic antigen family of proteins [42], F-box only protein 50, and glial fibrillary acidic protein, which may have regulatory roles in immune phenotypes of PCa [43] as well as cellular retinoic acid-binding protein 1 (CRABP1), which is implicated in PCa progression [44].

In addition, shared proteins in the U-EV proteomes for the two PCa groups included several keratins (type I cytoskeletal 16, KRT6A, and keratin, type II cytoskeletal 78), mutant haemoglobin alpha 2 globin chain, albumin, junction plakoglobin, Desmoglein-1, Glyceraldehyde-3-phosphate dehydrogenase, Annexin A2—a prognostic marker of aggressive PCa [45], Dermcidin (which is associated with proliferation and survival in PCa and a potential treatment target in PCa [46]), Hornerin—which has been highlighted as a urine biomarker for PCa [47], and SERPINB12 protein which is associated with proliferation and metastasis in lung cancer [48].

Pathway enrichment analysis was carried out to identify possible functional differences associated with the U-EV proteomes of PCa patients, compared with healthy controls, as well as comparing U-EV proteomes of lower and higher PCa Gleason scores. Gene ontology, KEGG and Reactome pathways, and disease–gene associations were assessed, and the results revealed considerable differences in the PCa U-EVs. Pathway enrichment analysis of the U-EV proteomes further highlighted considerable differences in immune, metabolic, and cancer-related pathways in the U-EV proteomic signatures of the PCa groups. Our findings correlate with other studies which have reported metabolic, inflammatory, and immune-related alterations in U-EVs associated with the pathogenesis and progression of PCa [49,50].

The GO pathways identified to be shared for both PCa groups (GS 6–7 and GS 8–9) were related to cadherin binding, protein binding, cell adhesion, S100 protein binding, and desmosome. KEGG pathways identified as shared for both PCa groups were *S. aureus* infection and oestrogen signalling, which is involved in PCa cell metabolism, proliferation, and disease progression [51].

The GO pathways enriched for the proteome of the GS 6–7 U-EVs highlighted roles in cytoskeletal function, cellular function, cellular communication, and immunity, and included the following: intermediate filament cytoskeleton organisation; organelle organisation; cellular component organisation; secretory vesicle; cytoplasmic vesicle membrane; apical plasma membrane; plasma membrane region; cell–cell contact zone; midbody; nucleus; intracellular organelle; and killing by host of symbiont cells. This highlights important roles of cytoskeletal behaviour and nuclear integrity for PCa [52] and links to the discussion on some of the individual proteins above.

The GO pathways enriched in the GS 8–9 U-EVs only included some key immune- and stress-related pathways, including the following: response to stress; humoral immune response; antimicrobial humoral response; defence response; defence response to other organisms; immune response; response to other organisms; cell killing; response to wounding; defence response to bacterium; fibrinolysis; platelet aggregation; response to oxidative stress; antioxidant activity; response to reactive oxygen species; response to biotic stimulus; response to toxic substance; positive regulation of receptor-mediated endocytosis lysosome; and proteasome complex. This highlights the complex roles of the immune microenvironment in PCa [53]. Several homeostatic and cellular communication processes were furthermore associated with the GS 8–9 U-EV proteomes, including the following: regulation of body fluid levels; homotypic cell–cell adhesion; cell adhesion; actin filament; postsynaptic actin cytoskeleton; focal adhesion; cell junction; membrane-bounded organelle; cellular oxidant detoxification; negative regulation of peptidase activity; regulation of peptidase and endopeptidase activity; enzyme inhibitor activity; cell–cell junction organisation; cell–cell junction assembly; negative regulation of endopeptidase activity; catabolic process; cellular catabolic process; hydrogen peroxide catabolic process; organic substance catabolic process; regulation of endopeptidase activity; multicellular organismal process; desmosome organisation; protein localisation to cell–cell junction; and positive regulation of gene expression. These associated GO pathways, which are linked to the diverse proteins identified in the U-EVs of the GS 8–9 patients, also reflect various metabolic adaptions in PCa [54].

KEGG pathways unique to the GS 8–9 U-EVs were related to gastric acid secretion, proteasome, apoptosis, oxytocin signalling pathway, arrhythmogenic right ventricular cardiomyopathy, and several neurodegenerative diseases (Parkinson’s disease, amyotrophic lateral sclerosis, prion disease, Alzheimer’s disease, and spinocerebellar ataxia), which may be of interest in relation to recent reports linking these conditions [55,56] and possible common therapeutic targets [57], or possibly linked to perineural invasion, associated with more aggressive PCa [58,59]. In addition, African trypanosomiasis was associated with the U-EV proteome of the GS 8–9 group, which may be of interest, as repurposing of melarsoprol and suramin showed some promise for PCa treatment [60,61].

There were 102 Reactome pathways enriched for the U-EV proteome of the GS 8–9 patients and related to stress and immune responses, mRNA stability, signalling cascades (including interleukins, which are a known risk factor for cancer [62]), the cell cycle and angiogenesis—including VEGF, which is associated with tumour grade, metastasis, and prognosis [63]. The top 25 scoring pathways based on strength of signal for the GS 8–9 U-EV proteomes included the following: scavenging of haeme from plasma, ER-Phagosome pathway, proteasome-mediated degradation, cellular response to chemical stress, apoptotic cleavage of cell adhesion proteins, and apoptosis, which are crucial target pathways for the intact apoptosis machinery of prostate cancer cells [64]. Within the top 25 pathways was also negative regulation of NOTCH4 signalling—which is associated with PCa disease progression and prognosis [65], NFE2L2-mediated nuclear events, regulation of mRNA stability, G2/M progression, MAPK6/MAPK4 signalling—which is an important player in cancer development [66], BTRC:CUL1-mediated degradation of NFE2L2, and GSK3B—which has been highlighted as a treatment target in PCa [67]. Additional top 25 scoring pathways were regulation of RUNX3 expression and activity, which plays a role in metastasis and angiogenesis in PCa [68]; translocation of GLUT4 to the plasma membrane; platelet degranulation; the recycling pathway of L1; and regulation of AURKA during mitotic entry and in early mitosis by FBXL7, with AURKA identified as a critical gene for PCa progression [69,70]. The Reactome pathways associated with the U-EV proteome of the GS 8–9 group therefore reflect some aspects of the complex microenvironment of PCa with relation to immune, vascular, and stromal associations [71].

Disease–gene association terms enriched in the GS 6–7 U-EV proteomes were related to stomach cancer, duodenal ulcer, and atrophic gastritis, which may reflect some risks associated with metastasis [72] or treatment of side effects and pathogenesis [73]. In the GS 8–9 U-EV proteomes, unique terms were related, among others, to skin cancer, lymphoma autoimmune disease, respiratory disease (which included COVID-19 [74]), primary immunodeficiency disease, allergic disease, liver disease [75,76], systemic mycosis [77], and proliferative glomerulonephritis, which is associated with increased cancer risk [78,79].

There has been a recent interest in using U-EVs and their various cargoes as disease-related biomarkers [80]. This includes recent assessment of U-EV-associated proteome signatures related to prostate tumours [10,12] and the identification of indicative PCa-associated micro-RNA and RNA U-EV cargoes [81,82,83,84,85]. However, results to date remain inconclusive and highlight the need for further research in the field of U-EV biomarker discovery for PCa. While limitations of our current study include relatively low patient numbers and grouping of GS 6–7 and GS 8–9, our findings do indicate some considerable differences in the proteomic U-EV profiles associated with PCa. Future studies will need to further assess interference from other physiological conditions and comorbidities to validate the robustness of U-EVs as clinical biomarkers for PCa in larger patient cohorts.

In summary, our findings showed significant differences in proteomic cargoes in U-EVs isolated from the urine of PCa patients compared to U-EVs from healthy controls, contributing to our current understanding of U-EVs for diagnostic use. Our findings therefore highlight the potential of U-EV proteome signatures as indicative biomarkers of PCa stage and may be translatable to clinical practice.

## 4. Materials and Methods

### 4.1. Urinary Samples from Patients

For this study (IRAS project ID: 29934, REC reference: 21/PR/0819, sponsored by East & North Hertfordshire Hospitals NHS Trust), urinary samples 65 ± 18 mL were collected from healthy controls (n = 20) and prostate cancer patients with Gleason scores 6–9 (n = 19); Table 1. This study was approved by the Institutional Review Board (or Ethics Committee) of the Bristol Research Ethics Community Centre (IRAS ID: 299344 approved in June 2021 and Local Research and Development Reference RD2019-34 in May 2021).

### 4.2. Isolation and Characterisation of Extracellular Vesicles from Urine

**Figure 5:**
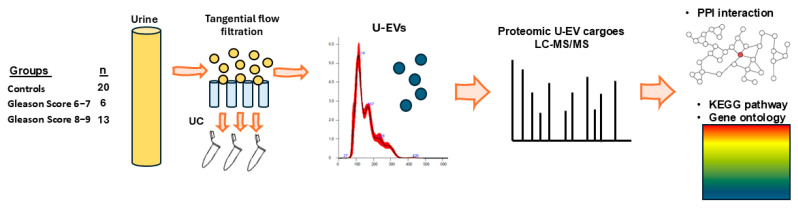
Workflow summary. Urine was concentrated by tangential flow filtration (TFF), U-EVs were further isolated by ultracentrifugation (UC) and characterised, and U-EV cargoes were then analysed by LC-MS/MS for proteomic cargoes. Proteomic datasets were analysed for protein–protein interactions (PPIs), KEGG pathways, and gene ontology, comparing controls to PCa groups of Gleason scores 6−7 and 8−9, respectively.

The urine was concentrated by tangential flow filtration (TFF) before proceeding to further EV isolation by ultracentrifugation (UC) (Figure 5). TFF was carried out as follows: an ultrafiltration disc 500 kDa molecular weight cutoff membrane (Ø44.5 mm, Biomax Polyethersulfone, Merck, Hoddesdon, Hertfordshire, UK) was mounted into a sterile, Teflon, filtration cassette, and the device was connected to a pressure regulator (Elvesys, Elveflow Microfluidics, Paris, France) using polymer tubing (Tub PEEK, 1/16″ od, Cole-Parmer Instrument Company Ltd., St. Neots, UK). A constant air pressure of 5 psi was applied at the headspace of the original 50 mL Falcon tubes containing the samples, and the urine was driven into the TFF. Under these conditions, the volumetric flow rate on the retentate side was 1 mL/s. Both retentate and permeate fractions were collected; only the retentate was recycled through the device until a 5 mL volume of sample was reached. To purify the samples further, 15 mL of 1 × PBS was added, and the filtration continued until a final volume of 1.5 mL was reached. A total of 250 µL of glycerol was added to each tube, and the samples were stored at −20 °C till further processing.

For downstream EV isolation following TFF, 1 mL of concentrated urine samples from TFF were used per individual. EVs were isolated by differential ultracentrifugation according to methods previously standardised and published in our laboratory, adhering to MISEV recommendations [86]. The concentrated urine samples from TFF were first centrifuged at 4000× *g* for 20 min at 4 °C, to discard any aggregates. The supernatants were then moved into fresh Eppendorf tubes and centrifuged at 100,000× *g* for 1 h at 4 °C. The resulting EV pellet was kept, discarding the supernatant, and 500 µL of DPBS was added to wash the enriched EV pellet, followed by a second centrifugation for 1 h at 100,000× *g*, at 4 °C. The final EV pellets were each resuspended in 100 µL of DPBS and assessed by nanoparticle tracking analysis (NTA) for quantification and size profiling, by transmission electron microscopy (TEM), and for surface marker detection by Western blotting and dSTORM imaging. Analysis of U-EV cargoes was performed for total proteomic cargoes by LC-MS/MS. The experimental workflow and workflow are shown in Figure 5.

#### 4.2.1. Nanoparticle Tracking Analysis

EVs were quantified, and size profiles were generated using the NS300 Nanosight (Malvern Panalytical, Malvern, UK) for nanoparticle tracking analysis (NTA), using a 488 nm blue laser, with the syringe pump speed set at 50 and the camera level set at 12 for capture. Samples were recorded for 4 × 60 s, and histograms were generated by averaging the four readings per sample; the threshold was set at level 5 for post-analysis processing. The NTA reports were used to identify the quantity of EVs/mL, modal size, and proportion of EV subpopulations based on size: small EVs (sEVs, ≤100 nm), medium-sized EVs (mEVs, 101–200 nm), and large EVs (lEVs, >200 nm).

#### 4.2.2. Western Blotting for EV Surface Markers

Two EV-specific markers, CD63 and flotillin-1, were assessed by dot blot and Western blotting for the U-EVs, and a positive signal was verified. In brief, samples were prepared in 2× reducing Laemmli sample buffer (BioRad, Watford, UK; containing 5% β-mercaptoethanol, Sigma-Aldrich, Gillingham, Dorset, UK) and boiled for 5 min at 100 °C. Samples were run by SDS-PAGE (4–20% TGX gels, BioRad) at 165 V for 52 min and transferred to nitrocellulose membranes using semi-dry transfer (1 h at 15 V), followed by blocking in 5% bovine serum albumin (BSA, Sigma-Aldrich) in TBS-T for 1 h at room temperature (RT). Primary antibody incubation with CD63 (ab216130, Abcam, Cambridge, UK) and Flotillin-1 (ab41927) was carried out overnight at 4 °C on a shaking platform, followed by washing for 3 × 10 min in TBS-T and secondary antibody incubation for 1 h at RT using HRP-labelled anti-rabbit IgG (BioRad, diluted 1/3000 in TBS-T). The membranes were washed for 5 × 10 min in TBS-T and developed using enhanced chemiluminescence (ECL, Amersham Biosciences, Buckinghamshire, UK) and the UVP BioDoc-ITTM System (Thermo Fisher Scientific, Dartford, UK).

#### 4.2.3. Transmission Electron Microscopy

The U-EVs were imaged by transmission electron microscopy (TEM). For imaging preparation, EV pellets were resuspended in 100 mM sodium cacodylate buffer (pH 7.4). Approximately 3–5 μL of the EV suspension was applied to a glow-discharged TEM grid with a carbon support film. The sample was partially air-dried for around 10 min before the grid was placed onto a drop of fixative solution (2.5% glutaraldehyde (Agar Scientific Ltd., Stansted, UK) in 100 mM sodium cacodylate buffer, pH 7.4) for 1 min at RT. The grid was subsequently transferred across three drops of distilled water for washing, with excess water being removed using filter paper. Then, the grid was placed onto a drop of staining solution of 2% aqueous uranyl acetate (Agar Scientific Ltd., Stansted, UK) for 1 min, and any excess stain was removed with filter paper before air drying. TEM imaging of the EVs was conducted using a JEOL JEM 1400 microscope (JEOL, Tokyo, Japan) operated at 80 kV, with magnifications ranging from 30,000× to 60,000×. Digital images were captured using an AMT XR60 CCD camera (Deben, Bury Saint Edmunds, UK).

#### 4.2.4. Direct Stochastic Optical Reconstruction Microscopy (dSTORM) Imaging of U-EVs

Single-molecule localisation-based super-resolution microscopy imaging of U-EVs was carried out to visualise EV surface markers labelled with fluorophores. The ONI EV profiler 2 kit and ONI Nanoimager system were used for labelling and visualisation of U-EVs by dSTORM (ONI UK, Oxford, UK). Samples were prepared according to the manufacturer’s instructions (ONI), following the manual sample preparation protocol (EV Profiler 2), using tetraspanin trio (TT) capture for capturing U-EVs while EV visualisation was carried out by labelling with tetraspanin trio (anti-CD9 + CD63 + CD81 (561)) and a pan-EV marker (488). Imaging of EVs for Pan-EV and Tetraspanin Trio detection was carried out using the AutoEV imaging and CODI analysis protocol according to the manufacturer’s specifications (ONI).

### 4.3. Proteomic Cargo Analysis of U-EVs by Liquid Chromatography with Tandem Mass Spectrometry (LC-MS/MS)

Total protein extracts from U-EV pools (controls, GS 6–7 and GS 8–9) were prepared using RIPA+ buffer (Sigma-Aldrich, Gillingham, UK, containing 10% protease inhibitor cocktail, Sigma-Aldrich), incubated for 2.5 h on a continuously rotating platform at 4 °C. The samples were centrifuged at 16,000× *g* for 30 min at 4 °C, and the protein-containing supernatant was collected. Proteins were diluted in 2× Laemmli sample buffer (BioRad), run 0.5 cm into a 12% TGX gel (BioRad), and cut out as one gel band per group for in-gel digestion followed by LC-MS/MS, performed by Cambridge Proteomics (University of Cambridge, Cambridge, UK). In brief, automated LC-MS/MS analysis was carried out using a Dionex Ultimate 3000 RSLC nanoUPLC system (Thermo Fisher Scientific Inc., Waltham, MA, USA) in conjunction with a QExactive Orbitrap mass spectrometer (Thermo Fisher Scientific Inc., Waltham, MA, USA). Separation of peptides was performed by reverse-phase chromatography at a flow rate of 300 nL/min and a Thermo Scientific reverse-phase nano Easy-Spray column (Thermo Scientific PepMap C18, 2 mm particle size, 100A pore size, 75 mm i.d. × 50 cm length). Peptides were loaded onto a pre-column (Thermo Scientific PepMap 100 C18, 5 mm particle size, 100A pore size, 300 mm i.d. × 5 mm length) from the Ultimate 3000 autosampler with 0.1% formic acid for 3 min at a flow rate of 15 mL/min. After this period, the column valve was switched to allow elution of peptides from the pre-column onto the analytical column. Solvent A was water + 0.1% formic acid and solvent B was 80% acetonitrile, 20% water + 0.1% formic acid. The linear gradient employed was 2–40% B in 40 min. Further wash and equilibration steps resulted in a total run time of 60 min. The LC eluent was sprayed into the mass spectrometer using an Easy-Spray source (Thermo Fisher Scientific Inc.). The *m*/*z* values of all eluting ions were measured in an Orbitrap mass analyser, and data-dependent scans (selecting top 20) were employed for automatic isolation and generation of fragment ions using the HCD collision cell, measured using the Orbitrap analyser (ESI-ORBITRAP-HCD). Both singly charged ions as well as ions with unassigned charge states were excluded from selection for MS/MS. A dynamic exclusion window of 20 s was applied. The data were processed post-run using Protein Discoverer (version 2.1., Thermo Fisher Scientific Inc.), converted to mgf files, and submitted to Mascot (Mascot search algorithm; Matrix Science, London, UK). Searching for hits was carried out against the UniProt database *Homo_sapiens*_20221011 (226,953 sequences; 74,609,178 residues) with peptide and fragment mass tolerances, respectively, set at 20 ppm and 0.1 Da. The threshold value for significance was set at *p* < 0.05, and the peptide cutoff score was set at 34.

### 4.4. Protein–Protein Interaction (PPI) Networks and Functional Pathway Enrichment Analysis of U-EV Proteome Cargoes

Protein hits from the three U-EV groups were functionally annotated, and protein–protein interaction (PPI) networks were generated using STRING analysis (Search Tool for the Retrieval of Interacting Genes/Proteins; https://string-db.org/, accessed on 8 May 2025). Data for the pathway analysis of the protein networks were exported as STRING network images in png format. Excel files were exported for pathway enrichment analyses, including gene ontology (GO biological process, molecular function, and cellular component), Kyoto Encyclopaedia of Genes and Genomes (KEGG), Reactome, and disease–gene associations (DISEASES).

### 4.5. Statistical Analysis

For comparison of datasets from the three study groups, GraphPad Prism version 10 was used. One-way ANOVA was used with statistical significance regarded as *p* < 0.05. STRING analysis was carried out with medium confidence, and the maximum number of interactors was set for the first shell query proteins only (https://string-db.org/, accessed on 8 May 2025).

## 5. Conclusions

This study assessed EV profiles from urine samples of individuals affected by PCa at Gleason scores 6–9, compared to healthy controls. U-EVs were characterised and assessed for proteomic cargo content by LC-MS/MS analysis, showing significant increase in protein content of the U-EVs from the PCa patients, with higher protein content associated with higher Gleason scores. Assessment of U-EV proteome pathway enrichment analysis revealed considerable changes and enrichment in GO, KEGG, and Reactome pathways in the PCa U-EVs, relating to inflammatory, stress, metabolic, and pro-cancerous processes. This was furthermore more prominent in the U-EVs from the higher-Gleason-score group (GS 8–9), compared with GS 6–7. Our findings highlight U-EVs as biomarkers informing disease pathways in PCa patients and offer a non-invasive biomarker tool that can be further developed for clinical use.

## Figures and Tables

**Table 1:** Clinicopathological features of subjects providing urine samples (PCa patients and healthy controls). Samples were included from Gleason scores 6–9 (n = 19) and controls (n = 20). PCa diagnosis, GS, and age range are shown.

Sample	n	Prostate Cancer Diagnosis	Gleason Score (GS)	Age Range
Controls	20	NO	NA	55.15 (33, 65, 27, 30, 51, 54, 57, 39, 52, 50, 69, 49, 18, 79, 73, 84, 68, 76, 76, 53)
GS 6	1	YES	6	72
GS 7	5	YES	7	71.4 (65, 66, 71, 73, 82)
GS 8	9	YES	8	64.7 (67, 77, 60, 53, 69, 53, 73, 60, 70)
GS 9	4	YES	9	69.8 (65, 68, 78, 68)

## Data Availability

The data are contained within the article and Supplementary Materials.

## Supplementary Materials

The following supporting information can be downloaded at https://www.mdpi.com/article/10.3390/ijms26146895/s1.
