# Supplementary material for: Urinary Extracellular Vesicle Signatures as Biomarkers in Prostate Cancer Patients
Source: Int J Mol Sci. 2025 Jul 18;26(14):6895. doi: 10.3390/ijms26146895 (PMC12295355; doi:10.3390/ijms26146895)
Supplement: Supplementary file 1 [file ijms-26-06895-s001.zip › Supplementary Table S1.pdf]

Supplementary Table S1. Proteomic EV cargo analysis by LC MS/MS – protein hits are listed for the three sample group pools respectively, showing controls, GS 6-7 and GS 8-9 U-EV proteomes. Protein IDs and gene name are displayed. A tick (V) represents that the protein hit was identified in the respective sample group. Hits identified only in the GS 6-7 group are highlighted in green, while hits identified only in the GS 8-9 group are highlighted in purple.

**Protein ID**<br>***Gene name***	**Protein name**	**Ctrl**	**GS 6-7**	**GS 8-9**
P02768	Serum albumin	V		
A0A0M4FNU3<br>*ALDOA*	Fructose-bisphosphate<br>aldolase	V		
B4DRT4	Phosphatidylethanolamine-binding protein 1	V		
A0A024R7G2<br>*RAB3D*	Ras-related protein Rab-3	V		
Q2QD09<br>U3KPZ0<br>*TPI1*	Triosephosphate isomerase	V		
V9HWF4<br>B4DHM5	Phosphoglycerate kinase	V		
A0A024QYX2	G protein-coupled receptor, family C, group 5, member B	V		
A0A6Q8PFE4<br>*ACTB*	Actin, cytoplasmic 1	V	V	
P04259	Keratin, type II cytoskeletal 6B	V	V	
Q6ICS0<br>*ANXA11*<br>Q5T0G8	Annexin 11	V	V	
D3DWI6<br>*PSCA*	Prostate stem cell antigen	V	V	
A0A248RGE3<br>*RPS27A*	Ubiquitin-40S ribosomal protein S27a	V	V	
H3BMU1<br>B4DLP1<br>*IST1*	IST1 homolog	V	V	
P31151<br>*S10A7*	Protein S100-A7<br>S100A7	V		V
A0A024R5Z9<br>*PKM2*<br>V9HWB8	Pyruvate kinase	V		V
B2R4M6<br>B2R5H0	Protein S100	V		V
H6VRF8<br>*KRT1*	Keratin, type II cytoskeletal 1	V	V	V
A0A1B0GVI3<br>*KRT10*	Keratin, type I cytoskeletal 10	V	V	V
P35908<br>*KRT2*	Keratin, type II cytoskeletal 2 epidermal	V	V	V
P13647<br>*KRT5*	Keratin, type II cytoskeletal 5	V	V	V
X6RBG4<br>*B3KTU0*	Uromodulin	V	V	V
P02533<br>*K1C14*	Keratin, type I cytoskeletal 14	V	V	V
B2R6J2<br>*VIL2*	cDNA, FLJ92973, highly similar to Homo sapiens villin 2 (ezrin) (VIL2), mRNA	V	V	V
A0A087WVJ0	Mucin-1	V	V	V
H0Y8D1<br>*PRSS1*	Serine protease 1	V	V	V
A0A024R8N9<br>*GPRC5C*	G protein-coupled receptor, family C, group 5, member C	V	V	V

A0A024RAY2<br>*KRT18*	Keratin 18		V	
Q9NSB4<br>*KRT82*	Keratin, type II cuticular Hb2		V	
A0A804CBC2<br>BAIAP2L2	Brain-specific angiogenesis inhibitor 1-associated protein 2-like protein 2		V	
P05109<br>*S10A8*<br>*S100A8*	Protein S100-A8		V	
A0A075B6Z2<br>*TRAJ56*	T cell receptor alpha joining 56		V	
Q4W4Y1<br>*Drd4*	Dopamine receptor interacting protein 4		V	
A0A024R4F1<br>*HEL-S-17*	Phosphopyruvate hydratase		V	
A0A087WVQ9<br>*EEF1A1*	Elongation factor 1-alpha		V	
A0A0A0MRQ5<br>*PRDX1*	Peroxiredoxin-1		V	
A0A3B3ITN8	Retinoic acid-induced protein 3		V	
O00526<br>Q9UEE8<br>*UPK2*	Uroplakin-2		V	
A0A024QZ42<br>*PDCD6*	Programmed cell death protein 6		V	
A0A024R9E4<br>*MAL2*	Mal, T-cell differentiation protein 2		V	
A0A024R2C5<br>*VPS4B*	Vesicle-fusing ATPase		V	
P15924	Desmoplakin		V	
A0A1U9X8X5<br>*CDSN*	Corneodesmosin		V	
P35527	Keratin, type I cytoskeletal 9		V	
P08779<br>*KRT16*	Keratin, type I cytoskeletal 16		V	V
A0A0S2Z428	HCG2039812, isoform CRA_b, KRT6A		V	V
A0A0K2BMD8<br>*HBA2*	Mutant hemoglobin alpha 2 globin chain		V	V
A0A0C4DGB6<br>H0YA55	Albumin		V	V
B4DE59<br>A0A024R1X8<br>*JUP*	Junction plakoglobin		V	V
Q02413<br>*DSG1*	Desmoglein-1		V	V
Q2TSD0<br>*GAPDH*<br>V9HVZ4	Glyceraldehyde-3-phosphate dehydrogenase		V	V
Q8N1N4<br>K2C78<br>*KRT78*	Keratin, type II cytoskeletal 78		V	V
A0A024R5Z7<br>B2R657<br>B4DDZ4	Annexin A2		V	V
P81605-2<br>*DCD*	Dermcidin		V	V
Q86YZ3<br>*HRNR*	Hornerin		V	V
Q3SYB5<br>Q96P63-2<br>*SERPINB12*	SERPINB12 protein		V	V
A0A804GS07	Actin, cytoplasmic 2			V

ACTG1				
A0A5C2FSS3	IGL c1_light_IGKV3-20_IGKJ4			V
A0A5C2G3D4	IGL c3788_light_IGKV3-20_IGKJ4			V
Q6EZE9<br>*DEFA3*	Defensin			V
B4DKM4<br>*CEACAM7*	cDNA FLJ51760, highly similar to Carcinoembryonic antigen-related cell adhesion molecule 7			V
H6VRG2<br>*KRT1*	Keratin, type II cytoskeletal 1			V
P48668<br>*KRT6C*	Keratin, type II cytoskeletal 6C			V
P19012<br>*KRT15*	Keratin, type I cytoskeletal 15			V
Q04695<br>*KRT17*	Keratin, type I cytoskeletal 17			V
Q4LE79<br>*DSP*	DSP variant protein			V
B3VL17<br>*B3VL05*	Beta globin			V
Q1KLZ0	Actin, cytoplasmic 1			V
Q0IIN1<br>*KRT77*	Keratin 77			V
K2C8	Isoform 2 of Keratin, type II cytoskeletal 8			
B3KTV0	highly similar to HEAT SHOCK COGNATE 71 kDa PROTEIN			V
D0PNI1<br>*YWHAZ*<br>B0AZS6	Epididymis luminal protein 4			V
Q5T749<br>*KPRP*	Keratinocyte proline-rich protein			V
P05089-2<br>*ARG1*	Isoform 2 of Arginase-1			V
A0A384P5Q0	Catalase			V
B3KPS3<br>*TUBA1A*	Tubulin alpha chain			V
Q6KB66-3<br>*K2C80*	Isoform 3 of Keratin, type II cytoskeletal 80			V
B2CIS9	Caspase 14, apoptosis-related cysteine peptidase			V
A0A024R952	Plakophilin 1			V
A0A0B4J259<br>*LYZ*	Lysozyme C			V
Q5D862<br>FILA2<br>*FLG2*	Filaggrin-2			V
B4DTV0<br>*AHNAK*<br>Q8N274	Similar to Homo sapiens AHNAK nucleoprotein (desmoyokin)			V
Q5T750	Skin-specific protein 32			V
Q4KMZ1-3<br>*IQCC*	Isoform 3 of IQ domain-containing protein C			V
A0A481SHK9<br>*HBB*	Hemoglobin subunit beta			V
A4UCS6<br>*PRDX6*	Peroxiredoxin 6			V
A0A6Q8PF43<br>*HSPB1*<br>B4DL87	Heat shock protein beta-1			V
Q9NZT1	Calmodulin-like protein 5			V
Q9HB00	Desmocollin 1			V
E7DVW5	Fatty acid binding protein 5			V

A0A0A0MSI0<br>*PRDX1*	Peroxiredoxin-1			V
A0A494C0J7	TGc domain-containing protein			V
B4DF70	cDNA FLJ60461, highly similar to Peroxiredoxin-2			V
B0AZN7	cDNA, FLJ79477, highly similar to Protein-glutamine gamma-glutamyltransferase K			V
Q5K684	SCCA1/SCCA2 fusion protein			V
P47929<br>LGALS7B	Galectin-7			V
B4DNK4/P14618	Pyruvate kinase			V
A0A7P0TAI0<br>*HSPA5*	78 kDa glucose-regulated protein			V
D3DP13	Fibrinogen beta chain			V
I0B0K3<br>*FLG*	Truncated profilaggrin			V
A0A0K0K1H8<br>*HEL-S-71p*	Serotransferrin			V
Q14574-2<br>*DSC3*	Isoform 3B of Desmocollin-3			V
P0DOX5<br>*IGG1*	Immunoglobulin gamma-1 heavy chain			V
A0A090N7V5	Gamma-glutamylcyclotransferase			V
B4E216<br>*C3*	Complement C3			V
B4DXF3<br>*BLMH*	Bleomycin hydrolase			V
K7EPT8	Glial fibrillary acidic protein			V
A0A1B0GV23	Cathepsin D			V
P42357-2<br>*HAL*	Isoform 2 of Histidine ammonia-lyase			V
A0A140VJI7	Testicular tissue protein Li 61			V
A0A1U9X7W7	Epididymis secretory sperm binding protein			V
H0YJM8	Proteasome subunit beta type-5			V
A0JNT2<br>*KRT83*	KRT83 protein			V
Q6ZVX7	F-box only protein 50			V
P0DOX2	Immunoglobulin alpha-2 heavy chain			V
P27482	Calmodulin-like protein 3			V
Q8WVV4-1<br>*POF1B*	Isoform 1 of Protein POF1B			V
A0A140VK43	Proteasome subunit alpha type			V
P0DOY2<br>*IGLC2*	Immunoglobulin lambda constant 2			V
Q6IAT9	Proteasome subunit beta			V
Q8N7G1<br>*PNP*	Purine nucleoside phosphorylase			V
P00739-2<br>*HPR*	Isoform 2 of Haptoglobin-related protein			V
Q8IW75	Serpin A12			V
Q9Y3R4	Sialidase-2			V
G5EA35	Caspase recruitment domain-containing protein 18			V
0A024R6I7<br>*SERPINA1*	Alpha-1-antitrypsin			V
A0A384N6H1	Epididymis secretory sperm binding protein			V
A0A0S2Z3E8<br>*FGA*	Fibrinogen alpha chain isoform 2			V
P31947-2<br>*SFN*	Isoform 2 of 14-3-3 protein sigma			V
P17900	Ganglioside GM2 activator			V
O94915	Protein furry homolog-like			V
A0A1L1UHR1	Voltage-dependent anion-selective channel protein1			V

A2IDD5	Coiled-coil domain-containingcprotein 78			V
F1T0F7	Cellular retinoic acid-binding protein 1			V
Q6FHY3<br>*LOR*	LOR protein			V
L0R5A1<br>CSF2RB	Alternative protein CSF2RB			V
A0A140VK46	Proteasome subunit beta			V
Q9Y446-2<br>*PKP3*	Isoform PKP3b of Plakophilin 3			V
A0A384MR50	HUMAN F-actin-capping protein subunit beta			V
S4R471	Protein AMBP			V
D6RF35	Vitamin D-binding protein			V
Q9NZH8-2<br>*IL36G*	Isoform 2 of Interleukin-36 gamma			V
A0A2R8YFB7	TBCEL-TECTA readthrough			V
A0A8I5QJW6<br>*SWTD2*	[Histone H3]-lysine(36) N-trimethyltransferase			V
B4DHB3<br>*PGK1*	Phosphoglycerate kinase			V
