# Supplementary material for: Urinary Extracellular Vesicle Signatures as Biomarkers in Prostate Cancer Patients
Source: Int J Mol Sci. 2025 Jul 18;26(14):6895. doi: 10.3390/ijms26146895 (PMC12295355; doi:10.3390/ijms26146895)
Supplement: Supplementary file 1 [file ijms-26-06895-s001.zip › Supplementary Table S2.pdf]

Supplementary Table S2. Biological Process GO pathways identified for the U-EV proteomes of the three groups. A tick (V) indicates that the pathway was identified in the respective group. Pathways identified only in the GS 6-7 group are highlighted in green, while pathways identified only in the GS 8-9 group are highlighted in purple.

**Biological GO term description**	**CTRL**	**GL 6-7**	**GL 8-9**
Glycolytic process	V		
Canonical glycolysis	V		
Anatomical structure homeostasis	V		
Hexose metabolic process	V		
Intermediate filament cytoskeleton organization		V	
Organelle organization		V	
Cellular component organization		V	
Killing by host of symbiont cells		V	
Cellular process		V	
Epidermis development	V	V	V
Keratinocyte differentiation	V	V	V
Intermediate filament organization	V	V	V
Keratinization	V	V	V
Epithelial cell differentiation	V	V	V
Epithelium development	V	V	V
Supramolecular fiber organization	V	V	V
Cytoskeleton organization	V	V	V
Peptide cross-linking	V	V	V
Multicellular organismal homeostasis	V	V	V
Retina homeostasis	V	V	V
Skin development		V	V
Tissue development		V	V
Establishment of skin barrier		V	V
Cell-cell adhesion		V	V
Multicellular organismal water homeostasis		V	V
Cell differentiation		V	V
Anatomical structure development		V	V
Animal organ development		V	V
Regulation of body fluid levels			V
Homotypic cell-cell adhesion			V
Response to stress			V
Cell adhesion			V
Cellular oxidant detoxification			V
Negative regulation of peptidase activity			V
Antimicrobial humoral response			V
Defense response			V
Humoral immune response			V
Response to toxic substance			V
Regulation of peptidase activity			V
Cell-cell junction organization			V
Cell-cell junction assembly			V

Negative regulation of endopeptidase activity			V
Platelet aggregation			V
Regulation of endopeptidase activity			V
Defense response to other organism			V
Hydrogen peroxide catabolic process			V
Response to biotic stimulus			V
Multicellular organismal process			V
Response to oxidative stress			V
Response to reactive oxygen species			V
Immune response			V
Response to other organism			V
Catabolic process			V
Desmosome organization			V
Cellular catabolic process			V
Cell killing			V
Response to wounding			V
Response to bacterium			V
Defense response to Gram-negative bacterium			V
Fibrinolysis			V
Defense response to bacterium			V
Protein localization to cell-cell junction			V
Positive regulation of gene expression			V
Positive regulation of receptor-mediated endocytosis			V
Organic substance catabolic process			V
