# Supplementary material for: Urinary Extracellular Vesicle Signatures as Biomarkers in Prostate Cancer Patients
Source: Int J Mol Sci. 2025 Jul 18;26(14):6895. doi: 10.3390/ijms26146895 (PMC12295355; doi:10.3390/ijms26146895)
Supplement: Supplementary file 1 [file ijms-26-06895-s001.zip › Supplementary Table S6.pdf]

Supplementary Table S6. Reactome pathways identified for the U-EV proteomes of the three groups. A tick (V) indicates that the pathway was identified in the respective group. Pathways identified only in the GS 6-7 group are highlighted in green, while pathways identified only in the GS 8-9 group are highlighted in purple.

REACTOME term description	CTRL	GL 6-7	GL 8-9
Glycolysis	V		
Gluconeogenesis	V		
Metabolism of carbohydrates	V		
Formation of the cornified envelope	V	V	V
Innate Immune System	V	V	V
Neutrophil degranulation	V	V	V
Developmental Biology	V	V	V
Cell junction organization	V	V	V
Type I hemidesmosome assembly	V		V
Immune System			V
Scavenging of heme from plasma			V
Cellular response to chemical stress			V
Apoptosis			V
Regulation of mRNA stability by proteins that bind AU-rich elements			V
AUF1 (hnRNP D0) binds and destabilizes mRNA			V
Negative regulation of NOTCH4 signaling			V
Cellular responses to stress			V
Vesicle-mediated transport			V
Translocation of SLC2A4 (GLUT4) to the plasma membrane			V
Apoptotic cleavage of cell adhesion proteins			V
MAPK family signaling cascades			V
Platelet degranulation			V
Hemostasis			V
The role of GTSE1 in G2/M progression after G2 checkpoint			V
Nuclear events mediated by NFE2L2			V
RAF/MAP kinase cascade			V
ER-Phagosome pathway			V
Signaling by Interleukins			V
MAPK6/MAPK4 signaling			V
Cross-presentation of soluble exogenous antigens (endosomes)			V
Regulation of activated PAK-2p34 by proteasome mediated degradation			V
Regulation of ornithine decarboxylase (ODC)			V
Recycling pathway of L1			V
Interleukin-1 family signaling			V
UCH proteinases			V
G2/M Checkpoints			V
SCF-beta-TrCP mediated degradation of Emi1			V
Vpu mediated degradation of CD4			V

Vif-mediated degradation of APOBEC3G			V
Autodegradation of the E3 ubiquitin ligase COP1			V
Degradation of AXIN			V
Degradation of DVL			V
Hh mutants are degraded by ERAD			V
Post-translational protein modification			V
Ubiquitin Mediated Degradation of Phosphorylated Cdc25A			V
Ubiquitin-dependent degradation of Cyclin D			V
FBXL7 down-regulates AURKA during mitotic entry and in early mitosis			V
Regulation of RUNX3 expression and activity			V
GSK3B and BTRC:CUL1-mediated-degradation of NFE2L2			V
Post-translational protein phosphorylation			V
NIK-->noncanonical NF-kB signaling			V
SCF(Skp2)-mediated degradation of p27/p21			V
Dectin-1 mediated noncanonical NF-kB signaling			V
Degradation of GLI1 by the proteasome			V
Degradation of GLI2 by the proteasome			V
GLI3 is processed to GLI3R by the proteasome			V
Hedgehog off state			V
Defective CFTR causes cystic fibrosis			V
Autodegradation of Cdh1 by Cdh1:APC/C			V
Asymmetric localization of PCP proteins			V
Oxygen-dependent proline hydroxylation of Hypoxia-inducible Factor Alpha			V
Hedgehog ligand biogenesis			V
Platelet activation, signaling and aggregation			V
Activation of NF-kappaB in B cells			V
Adaptive Immune System			V
APC/C:Cdc20 mediated degradation of Securin			V
Regulation of RAS by GAPs			V
Regulation of Insulin-like Growth Factor (IGF) transport and uptake by Insulin-like Growth Factor Binding Proteins (IGFBPs)			V
Metabolism of proteins			V
Regulation of PTEN stability and activity			V
Sensory processing of sound by inner hair cells of the cochlea			V
Orc1 removal from chromatin			V
Cdc20:Phospho-APC/C mediated degradation of Cyclin A			V
CDK-mediated phosphorylation and removal of Cdc6			V
Regulation of RUNX2 expression and activity			V
APC/C:Cdh1 mediated degradation of Cdc20 and other APC/C:Cdh1 targeted proteins in late mitosis/early G1			V

RHO GTPases activate IQGAPs			V
C-type lectin receptors (CLRs)			V
Adherens junctions interactions			V
Degradation of beta-catenin by the destruction complex			V
FCERI mediated NF-kB activation			V
Detoxification of Reactive Oxygen Species			V
Hedgehog on state			V
Signaling by high-kinase activity BRAF mutants			V
Erythrocytes take up oxygen and release carbon dioxide			V
Antimicrobial peptides			V
MAP2K and MAPK activation			V
Downstream TCR signaling			V
Signaling by RAF1 mutants			V
VEGFA-VEGFR2 Pathway			V
RUNX1 regulates transcription of genes involved in differentiation of HSCs			V
Signaling by moderate kinase activity BRAF mutants			V
Paradoxical activation of RAF signaling by kinase inactive BRAF			V
Signaling downstream of RAS mutants			V
Formation of annular gap junctions			V
ABC-family proteins mediated transport			V
Gap junction trafficking			V
Erythrocytes take up carbon dioxide and release oxygen			V
Infectious disease			V
Regulation of localization of FOXO transcription factors			V
SARS-CoV-2 targets host intracellular signalling and regulatory pathways			V
Chk1/Chk2(Cds1) mediated inactivation of Cyclin B:Cdk1 complex			V
Assembly of the pre-replicative complex			V
Interleukin-1 signaling			V
Separation of Sister Chromatids			V
Sensory processing of sound by outer hair cells of the cochlea			V
Class I MHC mediated antigen processing & presentation			V
HSP90 chaperone cycle for steroid hormone receptors (SHR) in the presence of ligand			V
Deubiquitination			V
Activation of BAD and translocation to mitochondria			V
SARS-CoV-1 targets host intracellular signalling and regulatory pathways			V
