# Supplementary material for: Urinary Extracellular Vesicle Signatures as Biomarkers in Prostate Cancer Patients
Source: Int J Mol Sci. 2025 Jul 18;26(14):6895. doi: 10.3390/ijms26146895 (PMC12295355; doi:10.3390/ijms26146895)
Supplement: Supplementary file 1 [file ijms-26-06895-s001.zip › Supplementary Table S7.pdf]

Supplementary Table S7. Disease-gene associations identified for the U-EV proteomes of the three groups. A tick (V) indicates that the pathway was identified in the respective group. Pathways identified only in the GS 6-7 group are highlighted in green, while pathways identified only in the GS 8-9 group are highlighted in purple.

DISEASES term description	CTRL	GL 6-7	GL 8-9
Anemia	V		
Congenital hemolytic anemia	V		
Carbohydrate metabolic disorder	V		
Pneumonia	V		
Glucose metabolism disease	V		
Organ system benign neoplasm	V		
Cell type benign neoplasm	V	V	
Gastrointestinal system cancer	V	V	
Organ system cancer	V	V	
Disease of cellular proliferation	V	V	
Stomach carcinoma	V	V	
Stomach cancer		V	
Duodenal ulcer		V	
Atrophic gastritis		V	
Skin disease	V	V	V
Keratosis	V	V	V
Palmoplantar keratosis	V	V	V
Amyloidosis	V	V	V
Autosomal dominant disease	V	V	V
Primary cutaneous amyloidosis	V	V	V
Familial visceral amyloidosis	V	V	V
Disease of anatomical entity	V	V	V
Skin carcinoma	V	V	V
Cutaneous T cell lymphoma	V	V	V
Acanthoma	V	V	V
Basal cell carcinoma	V	V	V
Immune system disease	V	V	V
Bullous congenital ichthyosiform erythroderma	V	V	V
Epidermolysis bullosa simplex Dowling-Meara type	V	V	V
Epidermolysis bullosa simplex with mottled pigmentation	V	V	V
Borst-Jadassohn intraepidermal carcinoma	V	V	V
Epidermolytic hyperkeratosis	V	V	V
Seborrheic keratosis	V	V	V
Mycosis fungoides	V	V	V
Carcinoma	V	V	V
Hepatocellular carcinoma	V	V	V
Epidermolytic acanthoma	V	V	V
Epidermolytic palmoplantar keratoderma	V	V	V
Pyelonephritis	V	V	V
Hematopoietic system disease	V	V	V

Genetic disease	V		V
Inherited metabolic disorder	V		V
Disease		V	V
Autosomal genetic disease		V	V
Pachyonychia congenita		V	V
Bullous skin disease		V	V
Nonepidermolytic palmoplantar keratoderma		V	V
Pemphigus		V	V
Epidermolysis bullosa		V	V
Arrhythmogenic right ventricular cardiomyopathy		V	V
Focal nonepidermolytic palmoplantar keratoderma		V	V
Steatocystoma multiplex		V	V
Hair disease		V	V
Naxos disease		V	V
Integumentary system disease			V
Dermatitis			V
Ichthyosis			V
Skin cancer			V
Disease by infectious agent			V
Monogenic disease			V
Ichthyosis vulgaris			V
Subcorneal pustular dermatosis			V
Autoimmune disease of skin and connective tissue			V
Respiratory system disease			V
Lung disease			V
Allergic rhinitis			V
Atopic dermatitis			V
Baraitser-Winter syndrome			V
Irritant dermatitis			V
Liver disease			V
Proliferative glomerulonephritis			V
Lymphoma			V
Protein-losing enteropathy			V
Eczema herpeticum			V
Food allergy			V
Respiratory allergy			V
Peanut allergy			V
Allergic disease			V
Primary immunodeficiency disease			V
Systemic mycosis			V
