# Supplementary material for: Urinary Extracellular Vesicle Signatures as Biomarkers in Prostate Cancer Patients
Source: Int J Mol Sci. 2025 Jul 18;26(14):6895. doi: 10.3390/ijms26146895 (PMC12295355; doi:10.3390/ijms26146895)
Supplement: Supplementary file 1 [file ijms-26-06895-s001.zip › Supplementary Tables S3-S5.pdf]

**Supplementary Table S3**. Molecular Function GO pathways identified for the U-EV proteomes of the three groups. A tick (V) indicates that the pathway was identified in the respective group. Pathways identified only in the GS 6-7 group are highlighted in green, while pathways identified only in the GS 8-9 group are highlighted in purple.

| Molecular GO term description | CTRL | GL 6-7 | GL 8-9 |
|---|---|---|---|
| Structural constituent of skin epidermis | V | V | V |
| Structural molecule activity | V | V | V |
| Structural constituent of cytoskeleton | V | V | V |
| Cell-cell adhesion mediator activity | | V | |
| Calcium-dependent protein binding | | V | |
| Cell adhesive protein binding involved in bundle of His cell-Purkinje myocyte communication | | V | |
| Cadherin binding | | V | V |
| Protein binding | | V | V |
| Cell adhesion molecule binding | | V | V |
| S100 protein binding | | V | V |
| Endopeptidase inhibitor activity | | | V |
| Identical protein binding | | | V |
| Enzyme inhibitor activity | | | V |
| Threonine-type endopeptidase activity | | | V |
| Antioxidant activity | | | V |
| Serine-type endopeptidase inhibitor activity | | | V |

**Supplementary Table S4**. Cellular component GO pathways identified for the U-EV proteomes of the three groups. A tick (V) indicates that the pathway was identified in the respective group. Pathways identified only in the GS 6-7 group are highlighted in green, while pathways identified only in the GS 8-9 group are highlighted in purple.

| Cellular component GO term description | CTRL | GL 6-7 | GL 8-9 |
|---|---|---|---|
| Extracellular exosome | V | V | V |
| Extracellular space | V | V | V |
| Cornified envelope | V | V | V |
| Vesicle | V | V | V |
| Secretory granule lumen | V | V | V |
| Secretory granule | V | V | V |
| Blood microparticle | V | V | V |
| Keratin filament | V | V | V |
| Supramolecular fiber | V | V | V |
| Polymeric cytoskeletal fiber | V | V | V |
| Cytosol | V | V | V |
| Cytoplasmic vesicle | V | V | V |
| Collagen-containing extracellular matrix | V | V | V |
| Cytoskeleton | V | V | V |
| Cell periphery | V | V | V |
| Intracellular non-membrane-bounded organelle | V | V | |
| Azurophil granule | V | V | |
| Intermediate filament cytoskeleton | | V | |

| | | | |
|---|---|---|---|
| Secretory vesicle | | V | |
| Membrane | | V | |
| Cytoplasmic vesicle membrane | | V | |
| Apical plasma membrane | | V | |
| Nucleus | | V | |
| Plasma membrane region | | V | |
| Cell-cell contact zone | | V | |
| Midbody | | V | |
| Intracellular organelle | | V | |
| Extracellular region | | V | V |
| Intermediate filament | | V | V |
| Desmosome | | V | V |
| Azurophil granule lumen | | V | V |
| Cytoplasm | | V | V |
| Cell-cell junction | | V | V |
| Plasma membrane | | V | V |
| Adherens junction | | V | V |
| Organelle | | V | V |
| Endomembrane system | | V | V |
| Extrinsic component of membrane | | V | V |
| Fascia adherens | | V | V |
| ficolin-1-rich granule membrane | | V | V |
| ficolin-1-rich granule lumen | V | | V |
| ficolin-1-rich granule | | | V |
| Vacuolar lumen | | | V |
| Supramolecular complex | | | V |
| Tertiary granule | | | V |
| Keratohyalin granule | | | V |
| Lysosome | | | V |
| Proteasome core complex | | | V |
| Endopeptidase complex | | | V |
| Proteasome complex | | | V |
| Anchoring junction | | | V |
| Proteasome core complex, beta-subunit complex | | | V |
| Focal adhesion | | | V |
| Cell junction | | | V |
| Membrane-bounded organelle | | | V |
| Tertiary granule lumen | | | V |
| Melanosome | | | V |
| Specific granule lumen | | | V |
| Postsynaptic actin cytoskeleton | | | V |
| Intracellular organelle lumen | | | V |
| Dense body | | | V |
| Intracellular anatomical structure | | | V |
| Actin filament | | | V |

**Supplementary Table S5**. KEGG pathways identified for the U-EV proteomes of the three groups. A tick (V) indicates that the pathway was identified in the respective group. Pathways identified only in the GS 6-7 group are highlighted in green, while pathways identified only in the GS 8-9 group are highlighted in purple.

| KEGG term description | CTRL | GL 6-7 | GL 8-9 |
|---|---|---|---|
| Glycolysis / Gluconeogenesis | V | | |
| Carbon metabolism | V | | |
| Estrogen signaling pathway | | V | V |
| Staphylococcus aureus infection | | V | V |
| Biosynthesis of amino acids | V | | V |
| Parkinson disease | | | V |
| Amyotrophic lateral sclerosis | | | V |
| Gastric acid secretion | | | V |
| Prion disease | | | V |
| Proteasome | | | V |
| Alzheimer disease | | | V |
| Apoptosis | | | V |
| Spinocerebellar ataxia | | | V |
| Arrhythmogenic right ventricular cardiomyopathy | | | V |
| Oxytocin signaling pathway | | | V |
| African trypanosomiasis | | | V |
